# iTRAQ-Based Quantitative Proteomics Analysis of HeLa Cells Infected With *Chlamydia muridarum* TC0668 Mutant and Wild-Type Strains

**DOI:** 10.3389/fmicb.2019.02553

**Published:** 2019-11-07

**Authors:** Yingzi Wang, Emmanuel Wirekoh Arthur, Na Liu, Xiaofang Li, Wenjing Xiang, Asamoah Maxwell, Zhongyu Li, Zhou Zhou

**Affiliations:** ^1^Institute of Pathogenic Biology, Hengyang Medical College, University of South China, Hengyang, China; ^2^Hunan Provincial Key Laboratory for Special Pathogens Prevention and Control, Pathogenic Biology Institute, University of South China, Hengyang, China

**Keywords:** *Chlamydia muridarum* (*C. muridarum*), isobaric tags for relative and absolute quantitation (iTRAQ), quantitative proteomics, TC0668, infection

## Abstract

*Chlamydia muridarum*, an obligate intracellular pathogen, was used to establish a murine model of female upper genital tract infection by *Chlamydia trachomatis*. TC0668 in *C. muridarum* is a hypothetical chromosomal virulence protein that is involved in upper genital tract pathogenesis. The infection of mice with the *C. muridarum* TC0668-mutant (G216*) strain results in less pathological damage in the upper genital tract. In this study, an isobaric tags for relative and absolute quantitation (iTRAQ)-based quantitative proteomics analysis was performed to identify differentially expressed proteins between TC0668 wild-type (TC0668^wt^) and TC0668 mutant (TC0668^mut^) strains at 6, 12, 18, and 24 h post-infection (p.i.). Of the 550 proteins differentially expressed at 18 h p.i., 222 and 328 were up-regulated and down-regulated, respectively, inTC0668^mut^-infected cells. The expression of seven up-regulated proteins (encoded by SRPRB, JAK1, PMM1, HLA-DQB1, THBS1, ITPR1, and BCAP31) and three down-regulated proteins (encoded by MAPKAPK2, TRAFD1, and IFI16) from the iTRAQ analysis were validated using quantitative real-time (qRT)-PCR. The qRT-PCR results were consistent with those of iTRAQ. Gene Ontology (GO) and Kyoto Encyclopedia of Genes and Genomes (KEGG) pathway analyses revealed that the differentially expressed proteins primarily participated in inflammatory responses, fibrosis, metabolic processes, and complement coagulation cascades, and were mainly enriched in the phosphatidylinositol 3′-kinase (PI3K)/Akt, nuclear factor kappa-B (NF-κB), and other signaling pathways. Using western-blotting and immunofluorescence detection, significant differences in activation of the PI3K/Akt and NF-κB signaling pathways were observed between the TC0668^wt^- and TC0668^mut^-infected cells. Differentially expressed proteins linked with inflammation and fibrosis were used in a protein-protein interaction network analysis. The results suggest that TC0668 may play a pivotal role in *C. muridarum*-induced genital pathology by inducing inflammatory responses and fibrosis, which may involve the activation of the PI3K/Akt and NF-κB signaling pathways.

## Introduction

Chlamydiae are Gram-negative obligate intracellular prokaryotic pathogens affecting humans and animals (Bachmann et al., 2014; Bommana and Polkinghorne, 2019). Female lower genital tract infection with *Chlamydia trachomatis* can spread to the upper genital tract, causing inflammatory pathologies such as hydrosalpinx that result in infertility (Land et al., 2009; Rodgers et al., 2011). However, the pathogenesis of *C. trachomatis* infection remains unclear. Although it is unknown whether *Chlamydia muridarum* causes disease in humans, it can be used to study the immunobiology of chlamydial infection and investigate the mechanisms underlying the pathogenesis in the urogenital tract (Morrison and Caldwell, 2002; Shah et al., 2005; Cheng et al., 2007; Chen et al., 2010).

Recent advancements in the manipulation of *C. trachomatis* plasmids and transformation of *C. muridarum* have revealed that the plasmid-encoded genes *Pgp3* and *Pgp5* are important virulence factors in *C. muridarum* for the induction of hydrosalpinx (Liu et al., 2014; Huang et al., 2015). Kari et al. showed that infection with plasmid-deficient *C. trachomatis* is highly attenuated in non-human primates, which induced an anti-chlamydial immune response (Kari et al., 2011). Then a transcriptional profiling of plasmid-bearing and plasmid-deficient *C. trachomatis* infected HeLa cells was conducted to explore the role of chlamydial plasmid in the host cell inflammatory response to infection and immune avoidance (Porcella et al., 2015). Additionally, TC0668, a chromosome-encoded hypothetical protein, is an important upper genital tract pathogenicity factor of *C. muridarum*, as TC0668 mutant strains (TC0668^mut^) exhibit attenuated virulence, involving reduced pathology in mice, compared with TC0668 wild-type strains (TC0668^wt^) (Chen et al., 2015; Conrad et al., 2016). Nevertheless, the molecular mechanisms of the TC0668 protein in the pathogenesis of *C. muridarum* need further investigation.

As TC0668 is a newly discovered virulence protein, with unknown homologous proteins and pathogenic mechanisms, an effective technical approach is needed for comprehensive screening and analysis of related molecules and signaling pathways that may be involved in the pathogenesis. In recent years, proteomics is a frontier method for investigating complex biological functions, which provide appropriate targets for researching on novel molecular biomarkers. In particular, isobaric tags for relative and absolute quantitation (iTRAQ) is an advanced high-throughput quantitative proteomics technique with high sensitivity, that has been rapidly developed and widely used to investigate the pathogenesis of many infectious agents (Jézéquel et al., 2019; Wu et al., 2019; Zhou et al., 2019). In this study, iTRAQ-based quantitative proteomic technology was used to screen and analyze the differentially expressed proteins in HeLa cells infected with *C. muridarum* TC0668^wt^ and TC0668^mut^ strains. Therefore, an iTRAQ-based proteomics analysis of TC0668 from *C. muridarum* may clarify its role in chlamydial pathogenesis.

We aimed to describe the proteomics profile of human epithelial HeLa cells infected with TC0668^wt^ and TC0668^mut^ strains, a pair of isogenic clones. The two clones differed in *tc0668* genotypes. Briefly, TC0668^mut^ strain carries the TC0668 G216* mutation, with the glycine (GGA) codon at position 216 changing to a stop codon (TGA) of TC0668 protein, and the rest of genome and plasmid of the two isogenic clones are identical (Chen et al., 2015; Conrad et al., 2016). Our results suggest that TC0668 participates in the induction of molecular responses and may be involved in the signaling pathways that underlie chlamydial pathogenesis.

## Materials and Methods

### Chlamydial Organisms and Cell Culture Conditions

The *C. muridarum* TC0668 wild-type strain (TC0668^wt^) and mutant strain (TC0668^mut^) are different only in the presence or absence of the chromosomal gene *tc0668* and its function. G13.32.1 (retaining the wild-type *tc0668* genotype of *C. muridarum* Nigg3 strain), and G13.11.1 (with a non-sense mutation in *tc0668* resulting from a premature stop codon at the 216th codon position of TC0668 protein, defined as TC0668 G216*), a pair of isogenic clones, which had been subjected to plaque purification and deep whole-genome sequencing to ensure the genotype, were used as TC0668^wt^ and TC0668^mut^ strains in the experiments (Chen et al., 2015; Conrad et al., 2016). HeLa cells (human cervical carcinoma epithelial cells, CCL-2; American Type Culture Collection) were cultured in Dulbecco's modified Eagle medium (DMEM) supplemented with 10% fetal bovine serum (DMEM-10) at 37°C in 5% CO_2_. Six-well plates containing HeLa cells were infected with TC0668^wt^ or TC0668^mut^ (multiplicity of infection [MOI] = 1), and the cell lysates were then collected at 6, 12, 18, and 24 h post-infection (p.i.).

### Phase Detection of *Chlamydia* and TC0668 in Cells Infected With TC0668^wt^ or TC0668^mut^

To assess the quantity and quality of organisms in parallel experiments, immunofluorescence assays (IFAs) and quantitative real-time PCR (qRT-PCR) were used. For the IFAs, HeLa cells grown on coverslips in 24-well tissue culture plates containing DMEM-10 with cycloheximide were infected with either *C. muridarum* TC0668^wt^ or TC0668^mut^ (MOI = 1). Briefly, cell samples were fixed with 4% paraformaldehyde for 30 min at 37°C, followed by permeabilizing with 0.1% Triton-100 dissolved in phosphate-buffered saline (PBS) for 15 min. After washing and blocking, the cells were subjected to antibody and chemical staining. A rabbit anti-chlamydial polyclonal antibody and a goat anti-rabbit IgG conjugated to the 488 dye (green, Jackson Immuno Research Laboratories) were used to visualize chlamydial inclusions. A mouse anti-TC0668 antibody and a goat anti-mouse IgG conjugated to the Cy3 dye (red; Jackson Immuno Research Laboratories) were used to visualize natural TC0668 proteins, which were only present in the cells infected with the TC0668^wt^ strain (at 6, 12, 18, and 24 h p.i.). The nuclei were stained with 4',6-diamidino-2-phenylindole (DAPI). After incubation with primary and secondary antibodies for 1 h at 37°C and washing with PBS five times, observation and imaging were performed using an inverted fluorescence microscope.

qRT-PCR analysis was used to determine the copy number of target *Chlamydia* genes. *Chlamydia 16S rRNA* was used as the internal reference to control the changing in the number of pathogens during the life cycle. The *tc0668* gene expression level was also assessed in cells, while *pgp8*, a steadily expressed *Chlamydia* plasmid gene in the growth and proliferation, were regarded as the control and compared with *tc0668* (Ferreira et al., 2013). TRIZOL lysate (Invitrogen, Carlsbad, CA, USA) was added to extract total RNA in enzyme-free tubes, and then the same amount of templates was combined with the SYBR Green Premix with ROX Reference Dye (TIANGEN, Beijing, China). Assays were performed in using a LightCycle 96 Instrument (Roche, Basel, Switzerland). All samples were amplified as follows: 50°C for 5 min and 95°C for 10 min; followed by 40 cycles of 95°C for 15 s, 55°C for 20 s, and 72°C for 20 s. After amplification, data were analyzed using the 2^−ΔCt^ method. Primer sequences of amplified genes are shown in Table 1.

**Table 1:** Primer sequences for qRT-PCR.

**Gene**	**Size (bp)**	**Sense primer (5^**′**^-3^**′**^)**	**Anti-sense primer (5^**′**^-3^**′**^)**
*tc0668*	1227	ATGATGGAACCTTTACGTTTCGGT	CTAAAAGCCATAACTTAATCTAAAACCATAGT
*pgp8*	993	GTGGGTAAAGGGATTTTATCTTTGCA	TTATATTAGGGCCATCTTCTTTGAGGC
*16S rRNA*	123	TGCTACAATGGCCGGTACAATGAG	GCGATTACTAGCGACTCCGACTTC
SRPRB	161	ACTCTCCGTGTCTGGCTCTTCC	CCTAACCGCTGCTCTGTATTCACC
JAK1	154	TCCTGGTGCCTGTCTGCTTCC	CATCCTTGACATCACTGCCGACTG
PMM1	178	GCATGAGCCACCACGCACAG	GGAGAGCCAGAGCCAGACAGAG
HLA-DQB1	196	GTGGCGTTGGAGGCTTCGTG	TCCTGTCTCCTCGCACTTCTTCTC
MAPKAPK2	110	CGGATGGAGGAGGTCGGATGG	CTGAGCCACCACCACCACTAATTC
IFI16	113	CAGGCAGCAAGTGAGCAGAGC	GACACCAGCACAGAAGCCACAG
TRAFD1	142	TGTGGTGGTAGGCTCCTGTAATCC	TTGAGACGGAGTTCGCTTCTGTTG
ITPR1	138	CAGTTGGCGTGAGACAATCAGGAG	AAGGCGGTGGAGGCAGTAGC
BCAP31	91	TACGACCGCTTGCTGGAGGAG	TGCCTCATCCTGCTCTGCTCTC
THBS1	81	CCAGATCAGGCAGACACAGACAAC	AGGCAGCACCTTACCGTCTCC

### Protein Extraction and iTRAQ Labeling

Infected cells were harvested and then lysed with SDT buffer (4% sodium dodecyl sulfate [SDS], 1 mM dithiothreitol [DTT], and 100 mM Tris-HCl). The lysate was sonicated, boiled for 15 min, and centrifuged at 14,000 × g for 40 min. The protein level in the supernatant was quantified with a Bicinchoninic Acid (BCA) Protein Assay Kit (Bio-Rad, CA, USA). iTRAQ assays were performed according to the manufacturer's instructions (Applied Biosystems; Ross et al., 2004).

### Strong Cation Exchange (SCX) and Nano Liquid Chromatography Coupled With Tandem Mass Spectrometry (LC-MS/MS) Analysis

iTRAQ labeled peptides were fractionated using SCX chromatography with an AKTA Purifier system (GE Healthcare; Huang et al., 2019; Qin et al., 2019). The dried peptide mixture was reconstituted and acidified with 2 mM buffer A and loaded onto a PolySULFOETHYL 4.6 × 100 mm column. Peptides were eluted at a flow rate of 1 mL/min with a gradient of 0% buffer B for 25 min, 0–10% buffer B during 25–32 min, 10–20% buffer B during 32–42 min, 20–45% buffer B during 42–47 min, 45–100% buffer B during 47–52 min, 100% buffer B during 52–60 min, and buffer B was reset to 0% after 60 min. The elution was monitored by absorbance at 214 nm, and 30 fractions were collected every 1 min. Collected fractions were distributed into 15 fractions and desalted on C18 Cartridges (Empore™ SPE Cartridges C18 (standard density), bed I.D. 7 mm, volume 3 mL, Sigma), concentrated by vacuum centrifugation, and reconstituted in 40 μL of 0.1% (v/v) trifluoroacetic acid.

Each fraction was then injected for nanoLC-MS/MS analysis, which was performed on a Q Exactive mass spectrometer (Thermo Scientific) that was operated in positive ion mode coupled to Easy nLC (Thermo Fisher Scientific) for 60 min. MS data were acquired using a data-dependent top 10 method. Automatic gain control (AGC) target was set to 1e6, and maximum inject time to 50 ms. Dynamic exclusion duration was 60.0 s. Survey scans were acquired at a resolution of 70,000 at m/z 200 and resolution for HCD spectra was set to 17,500 at m/z 200, and isolation width was 2 m/z. Normalized collision energy was 30 eV and the underfill ratio, which specifies the minimum percentage of the target value likely to be reached at maximum fill time, was defined as 0.1%. The instrument was run with peptide recognition mode enabled.

### Proteomic and Bioinformatics Analysis

Protein identification and iTRAQ were performed with the Mascot (version 2.2; Matrix Science, Massachusetts, USA) and Proteome Discoverer software (version 1.4; Thermo Scientific, Massachusetts, USA) against the UniProt Human Database (133549 sequences, downloaded on March 3rd, 2013). The parameters used included: mass tolerance = 20 ppm, MS/MS tolerance = 0.1 Da, enzyme = trypsin, missed cleavage = 2, oxidation (M), iTRAQ 8plex (Y) as the potential variable modifications, and carbamidomethyl (C), iTRAQ8plex (N-term), iTRAQ 8plex (K) as the fixed modifications. Decoy database search was used to calculate the false discovery rate (FDR) for peptide identification, using a screening criterion of FDR ≤ 0.01. Differential protein expression was defined as an iTRAQ ratio between the two groups of > 1.2 or < 0.83. All the differentially expressed proteins of the proteomics were analyzed via UniProt (http://www.uniprot.org/).

Venn diagram was constructed to analyze the common differentially expressed proteins between two strains at 6, 12, 18, and 24 h p.i. Gene Ontology (GO) analysis (version go_201608.obo; www.geneontology.org) was used to evaluate the biological significance of the differentially expressed proteins. GO analysis provide controlled vocabularies for the description of the biological process, molecular function, and cellular component of gene products by organism databases, and the differentially expressed proteins that are involved in similar processes, function and components were divided into corresponding clusters. Kyoto Encyclopedia of Genes and Genomes (KEGG) pathway analysis was performed to investigate potential biological pathways using online software (KEGG Automatic Annotation Server [KAAS]). Information on protein–protein interactions (PPIs) of the studied proteins was retrieved using IntAct software (http://www.ebi.ac.uk/intact/main.xhtml) and Search Tool for the Retrieval of Interacting Genes/Proteins software (STRING; http://string-db.org/). The results were imported into Cytoscape5 software (version 3.2.1; RT-https://cytoscape.org/) to visualize and analyze the functional PPI networks.

### qRT-PCR Validation of Proteomics Results

To validate the proteomics results, qRT-PCR analysis was performed to determine the mRNA expression levels of 10 differentially expressed proteins (with the corresponding gene names SRPRB, JAK1, PMM1, HLA-DQB1, THBS1, ITPR1, BCAP31, MAPKAPK2, TRAFD1, and IFI16) at random. Primer sequences of amplified genes are shown in Table 1. The general qRT-PCR steps were the same as described above. Total RNAs of TC0668^wt^ and TC0668^mut^ were collected at 18 h p.i. *Chlamydia 16S rRNA* was used to normalize the mRNA levels of the target genes. Samples were amplified as follows: 50°C for 5 min and 95°C for 10 min; followed by 45 cycles of 95°C for 5 s, 57°C for 10 s, and 72°C for 20 s.

### Validation of KEGG Analysis

Western blotting and IFA were used for validation of the KEGG analysis. For western blotting, proteins from TC0668^wt^- and TC0668^mut^-infected cells at 6, 12, 18, and 24 h p.i were obtained as follows. First, the cells in each well (six-well plate) were added with 100 μL radioimmunoprecipitation assay (Solarbio, Beijing, China) lysis buffer, 1 μL of the protease inhibitor phenylmethylsulfonyl fluoride (Solarbio, Beijing, China) and phosphatase inhibitor cocktail (CWBIO, Beijing, China) for phospho-Akt (p-Akt). The cells lysate were removed to 2 mL Eppendorf tubes on ice and then centrifuged at 15,294 × g at 4°C for 10 min. The supernatants were analyzed using 12% SDS-polyacrylamide gel electrophoresis (PAGE), and then transferred to a polyvinylidene fluoride (PVDF) membrane (Millipore, Billerica, MA, USA). The membrane was blocked using 5% skim milk with Tris-buffered saline plus Tween 20 (TBST) buffer for 2 h, and then incubated with rabbit raised monoclonal PI3K, Akt, p-Akt, p53, and NF-κB (p65) antibodies (all from CST, Danvers, MA, USA) at 37°C for 1 h. The membrane was incubated with a horseradish peroxidase (HRP)-conjugated goat anti-rabbit IgG secondary antibody (Sigma-Aldrich, St. Louis, Missouri, USA) at 37°C for 1 h. Visualization was conducted with an Enhanced Chemiluminescence (ECL) Kit (Santa Cruz Biotech, Santa Cruz, CA, USA). All proteins were normalized to the glyceraldehyde 3-phosphate dehydrogenase (GAPDH) control after the belts were scanned by gel quantification software (ImageJ 2.0.0-rc-54, Java 1.8.0-66, https://imagej.net/Downloads), and the relative intensity of target protein was calculated finally.

To further demonstrate the translocation of NF-κB (p65) into cell nuclei, IFAs were performed with TC0668^wt^- and TC0668^mut^-infected HeLa cells at 6, 12, 18, and 24 h p.i. The primary antibody was rabbit raised monoclonal p65 (CST) antibody, the secondary antibody was goat anti-rabbit IgG conjugated to the 488 dye (green; Jackson Immuno Research Laboratories), and DAPI dye core (blue) was used to label cell nuclei. Visualization and imaging (inverted fluorescence microscopy) were performed as mentioned above.

### Statistical Analysis

Data on mRNA levels were analyzed using the 2^−ΔCt^ method, and the data obtained by western blotting in three independent experiments were analyzed using one-way analysis of variance [ANOVA]. Experimental data were all statistically analyzed using SPSS software (version 13.0; SPSS, Chicago, IL, USA). Significant A (including *t*-tests and one-way ANOVA) was performed to analyze the experimental data of proteomics. A *P*-value < 0.05 was considered statistically significant.

## Results

### Equal Numbers of Inclusions but Not TC0668 Were Detected in TC0668^wt^- and TC0668^mut^-Infected Cells

**Figure 1:**
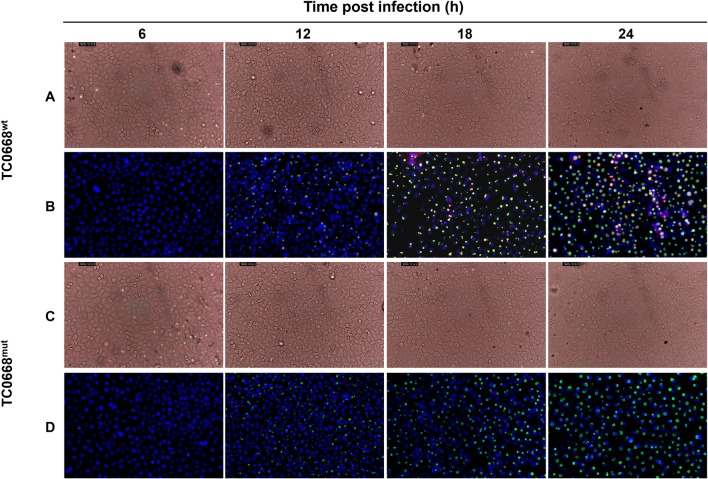
Indirect immunofluorescence assay (IFA) of HeLa cells infected with *C. muridarum* TC0668^wt^ or TC0668^mut^ strains. With 2.5 × 10^5^ IFU/well inoculum (MOI = 1), *Chlamydia*-infected HeLa cells were photographed using optical microscopy at 6, 12, 18, and 24 h p.i. Chlamydial inclusion bodies (green) are visible in both TC0668^wt^- and TC0668^mut^-infected cells, whereas the TC0668 protein (red) is only visible in TC0668^wt^-infected HeLa cells. Magnification, ×200. TC0668^wt^-infected HeLa cells were photographed by using phase contrast microscopy **(A)** and IFA **(B)** at 6, 12, 18, and 24 h p.i. TC0668^mut^-infected HeLa cells were photographed by using phase contrast microscopy **(C)** and IFA **(D)** at 6, 12, 18, and 24 h p.i.

HeLa cells infected with *C. muridarum* TC0668^wt^ and TC0668^mut^ strains were imaged at 6, 12, 18, and 24 h p.i. using an optical microscope (Figure 1). At 12, 18, and 24 h p.i, the inclusions (green) of both strains were visualized, but no obvious green fluorescence was observed in either group at 6 h p.i. No differences in inclusion formation was observed between two strains at 12, 18, and 24 h p.i, whereas TC0668 (red) was only observed on the membrane of chlamydial elementary bodies (EBs) or reticulate bodies (RBs) in the TC0668^wt^ group and not in the TC0668^mut^ group at any detected time points.

**Figure 2:**
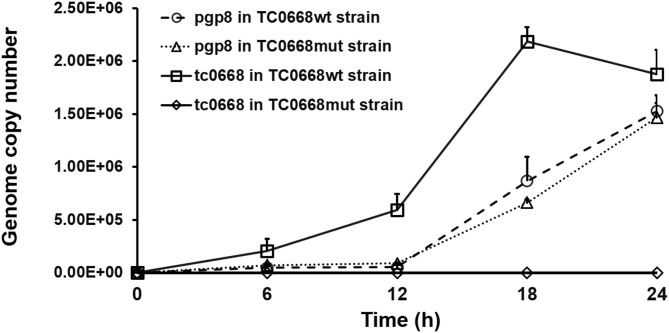
qRT-PCR analysis of *tc0668* gene copy number in *C. muridarum* TC0668^wt^- and TC0668^mut^-infected HeLa cells. With 1 × 10^6^ IFU/well inoculum (MOI = 1), the copy number of the *C. muridarum* gene *tc0668* was determined using qRT-PCR, and the *C. muridarum* plasmid gene *pgp8* was used as the control. *16S rRNA* was used to normalize *tc0668* and *pgp8* signals. Three biological replicates of each time point were performed, and points represent mean and standard errors. Copy number differences of *tc0668* between TC0668^wt^- and TC0668^mut^-infected cells were statistically significant (one-way ANOVA, *P* < 0.05).

qRT-PCR was used to detect target RNAs of *C. muridarum* TC0668^wt^ and TC0668^mut^ strains at 6, 12, 18, and 24 h p.i (Figure 2). *Pgp8*, an essential plasmid gene, is stably expressed during the chlamydial development cycle, and it was used as a control to evaluate *tc0668* expression at the transcriptional level. Both *tc0668* (*tc0668*/*16S rRNA*) and *pgp8* (*pgp8*/*16S rRNA*) were gradually upregulated from 0 to 18 h p.i and stabilized at 18 h p.i. Figure 2 illustrates that the *pgp8* gene copy numbers in the two groups were approximately equivalent. However, *tc0668* was not detected in the TC0668^mut^ group, while it was detected in the TC0668^wt^ group (one-way ANOVA, *P* < 0.05).

### Comparative Proteomics Analysis Using iTRAQ

**Figure 3:**
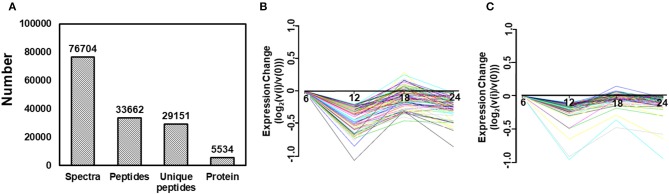
Quantitative proteomic analysis of HeLa cells infected with *C. muridarum* TC0668^wt^ or TC0668^mut^ strains. **(A)** Basic statistics of proteome results from iTRAQ. Spectra, secondary mass spectra after quality control; Unique peptides, identified peptides that belong to only a group of proteins; and protein, identified proteins using Mascot 2.3.02 software. **(B)** Trends of differentially expressed proteins in TC0668^wt^-infected cells at 6, 12, 18, and 24 h p.i. **(C)** Trends of differentially expressed proteins in TC0668^mut^-infected cells at 6, 12, 18, and 24 h p.i.

To identify the differentially expressed proteins between TC0668^wt^- and TC0668^mut^-infected cells, protein profiles of these two strains were compared at 6, 12, 18, 24 h p.i. As presented in Figure 3A, iTRAQ analysis of the TC0668^wt^- and TC0668^mut^-infected cell proteome showed 76,704 queries in the database, and resulted in 5,534 identified proteins, 33,662 peptides using Mascot software. According to the protein quantification criteria (cut-off values of 1.2-fold for up-regulation and 0.83-fold for down-regulation), compared to TC0668^wt^-infected cells, 265, 270, 222, and 197 up-regulated proteins and 306, 245, 328, and 214 down-regulated proteins (totaling 571, 515, 550, and 411 modulated proteins) were identified in the TC0668^mut^-infected cells at 6, 12, 18, and 24 h p.i., respectively. Figures 3B,C show the trends over time of differentially expressed proteins in TC0668^wt^ and TC0668^mut^-infected samples, respectively.

**Figure 4:**
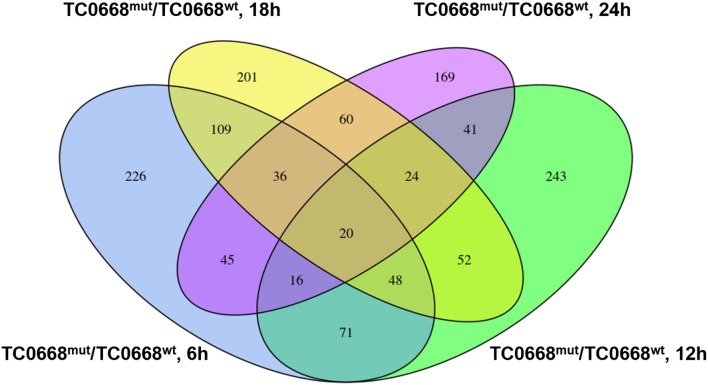
Four-way Venn diagram of the total number of proteins significantly differentially expressed (*P* < 0.05) between HeLa cells infected with *C. muridarum* TC0668^wt^ or TC0668^mut^ strains at 6, 12, 18, and 24 h p.i. Numbers of shared or unique proteins are indicated at the intersections of the circles in the Venn diagram.

A four-way Venn diagram was constructed to analyze the numbers of differentially expressed proteins between TC0668^wt^- and TC0668^mut^-infected cells (*P* < 0.05) that were unique or common to the different time points (Figure 4). Only 226, 243, 201, and 169 differentially expressed proteins were unique at 6, 12, 18, and 24 h p.i., respectively; 378 were shared by any two of the four time points. One hundred and twenty-four were shared by any three of the four time points; and 20 were common to all four time points.

The 20 differentially expressed proteins that were common to all four time points were analyzed using UniProt database. Two inflammation-related proteins were up-regulated (encoded by INPP5D) or down-regulated (encoded by MAPKAPK2) in TC0668^mut^-infected cells. Four proteins related to signaling pathways were up-regulated (encoded by INPP5D and MYO10) or down-regulated (encoded by ITGA6 and MAPKPKA2). Three proteins with binding functions were up-regulated (encoded by GNL3L) or down-regulated (encoded by CYB5R4 and CWC15) in TC0668^mut^-infected cells (Supplementary Materials).

### qRT-PCR Validation of Differentially Expressed Proteins

**Figure 5:**
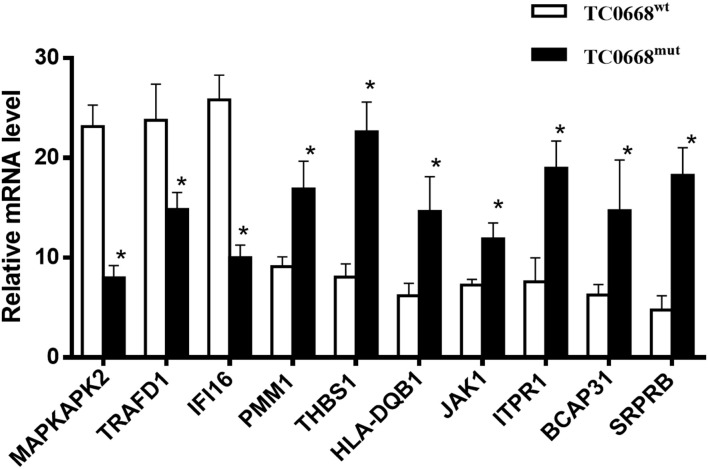
Validation of profiling data with qRT-PCR. mRNA expression levels of seven up-regulated (encoded by SRPRB, JAK1, PMM1, HLA-DQB1, BCAP31, ITPR1, and THBS1) and three down-regulated (encoded by MAPKAPK2, TRAFD1, and IFI16) proteins in *C. muridarum* TC0668^mut^-infected HeLa cells were determined using qRT-PCR at 18 h p.i, and compared with those of *C. muridarum* TC0668^wt^-infected group. mRNA levels from three replicates for each group are expressed as mean and the standard errors. *represents that all copy number differences between TC0668^wt^ and TC0668^mut^ were statistically significant (*t* test, *P* < 0.05).

To validate the iTRAQ-based proteomics results, qRT-PCR analysis was performed on 10 selected proteins that were differentially expressed between TC0668^mut^- and TC0668^wt^-infected cells. mRNA expression levels of seven up-regulated proteins (encoded by SRPRB, JAK1, PMM1, HLA-DQB1, THBS1, ITPR1, and BCAP31) and three down-regulated proteins (encoded by MAPKAPK2, TRAFD1, and IFI16) were validated using qRT-PCR. As shown in Figure 5, in TC0668^mut^-infected cells, the mRNA expression levels of the seven up-regulated proteins were significantly increased, whereas the mRNA expression levels of the three down-regulated proteins were significantly reduced (*P* < 0.05). Thus, the qRT-PCR results (regarding transcript levels) are consistent with those of the proteomics analysis.

### GO and KEGG Analyses of Differentially Expressed Proteins Between TC0668^wt^- and TC0668^mut^-Infected Cells

**Figure 6:**
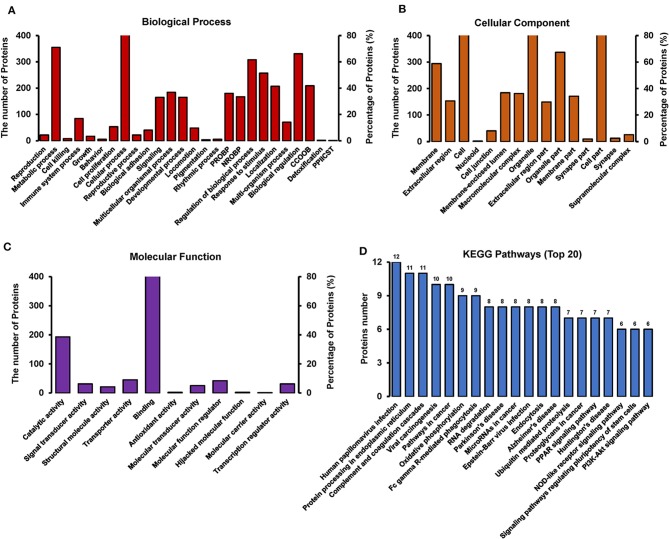
GO and KEGG pathway analysis of differentially expressed proteins between TC0668^wt^ and TC0668^mut^ strains at 18 h p.i (*P* < 0.05). **(A)** Biological process. The items “PROBP,” “NROBP,” “CCOOB,” and “PPIICST” are the abbreviations of “positive regulation of biological process,” “negative regulation of biological process,” “cellular component organization or biogenesis,” and “presynaptic procell involved in chemical synaptic transmission,” respectively. **(B)** Cellular component. The item “cell” including all related cell components besides the parallel cell part, membrane, nucleoid and so on. **(C)** Molecular function. **(D)** KEGG pathway analysis.

GO annotation of the 550 differentially expressed proteins at 18 h p.i. was analyzed using Blast2GO software. The analysis revealed the diversity of biological processes related to the differentially expressed proteins (Figure 6A), including cellular process, metabolic process, immune system process, and biological regulation. It also showed that these differentially expressed proteins are involved in a large number of molecular functions (Figure 6B), such as binding, catalytic activity, transporter activity, and molecular function regulation, as well as being associated with multiple cellular components (Figure 6C), including the cell, cell part, organelle, and membrane.

KAAS software was used to identify the potential biological pathways. The 550 differentially expressed proteins were allocated to 240 KEGG pathways. The pathways are mainly involved in oxidative phosphorylation, endocytosis, complement coagulation cascades, and the PPAR, PI3K-Akt, NOD-like receptor, and other signaling pathways (Figure 6D). Collectively, the findings provide relevant information to guide future research on the molecular basis of the role of TC0668 in *C. muridarum* pathogenesis.

### Western Blotting and IFA Validation of Proteomics Analysis

**Figure 7:**
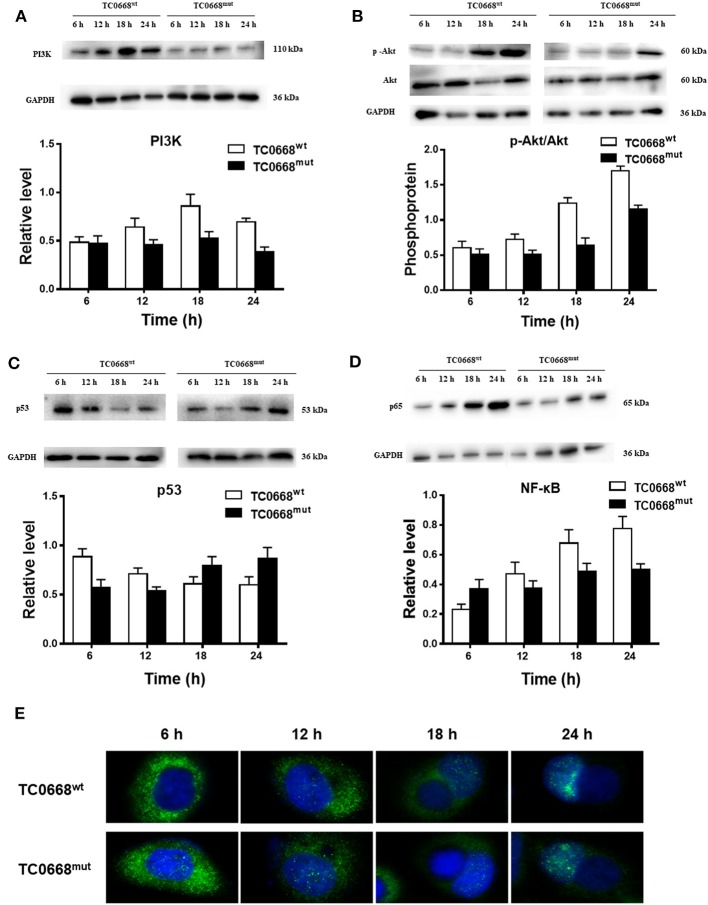
Activation of NF-κB and PI3K/Akt signal pathways as determined using western blotting and IFA. With 1 × 10^6^ IFUs/well inoculum (six-well plate) or 2.5 × 10^5^ IFUs/well inoculum (24-well plate), expression of PI3K, p-Akt, p53, and NF-κB (p65) representing activation of PI3K/Akt and NF-κB signal pathways were determined using western blotting or IFA, respectively. Gel quantification software was used to calculate the relative intensity of the corresponding signals. The relative value (target protein/GAPDH) differences of PI3K, p53, and NF-κB (p65) molecules and the relative expression level of p-Akt (p-Akt/Akt) between TC0668^wt^- and TC0668^mut^-infected cells were statistically significant (one-way ANOVA, *P* < 0.05). **(A)** Expression of PI3K molecules in TC0668^mut^- and TC0668^wt^-infected cells were determined using western blotting at 6, 12, 18, and 24 h post-infection. **(B)** Expression of p-Akt and total Akt in TC0668^mut^- and TC0668^wt^-infected cells were determined using western blotting at 6, 12, 18, and 24 h post-infection. **(C)** Expression of p53 molecules in the TC0668^mut^- and TC0668^wt^-infected cells at 6, 12, 18, and 24 h post-infection. **(D)** Expression of p65 molecules in the TC0668^mut^- and TC0668^wt^-infected cells at 6, 12, 18, and 24 h post-infection. **(E)** NF-κB molecules in the cytoplasm and nuclei of TC0668^mut^- and TC0668^wt^-infected HeLa cells at 6, 12, 18, and 24 h post-infection. DAPI dye core (blue), NF-κB fluorescence secondary antibody is 488 dye (green).

Based on the proteomics results, differentially expressed proteins were significantly enriched in several signaling pathways. We further verified the activation of the PI3K/Akt and NF-κB signaling pathways, which may be involved in the pathogenesis of chlamydial infections, based on the expression of PI3K, p-Akt, p53 (a tumor suppressor that can be downregulated by activation of PI3K/Akt pathway), and NF-κB (p65). As shown in Figure 7A, cells infected with *C. muridarum* TC0668^wt^ strains for 6, 12, 18, and 24 h displayed a significantly higher level of PI3K compared to TC0668^mut^-infected cells at all four time points (*P* < 0.05). The relative expression level of p-Akt (p-Akt/Akt) in the TC0668^wt^-infected cells was significantly higher than that in the TC0668^mut^-infected cells (*P* < 0.05) (Figure 7B). In contract, the level of p53 was significantly lower in the TC0668^wt^-infected cells compared to the TC0668^mut^-infected cells during chlamydial infection (*P* < 0.05) (Figure 7C).

As depicted in Figure 7D, NF-κB (p65) expression in TC0668^wt^-infected cells was significantly higher at all four time points than that in TC0668^mut^-infected cells (*P* < 0.05). It was noticeable that NF-κB (p65) expression was significantly increased in TC0668^wt^-infected cells at 18 h p.i. To further evaluate the role of the NF-κB signaling pathway in the TC0668 virulence mechanism, the colocalization of NF-κB molecules and the nuclei of cells infected with either the TC0668^wt^ or TC0668^mut^ strains at 6, 12, 18, and 24 h p.i were visualized using IFAs (Figure 7E). NF-κB molecules (green) in TC0668^wt^-infected cells were localized outside the nuclei (blue) at 6 h p.i, and became increasingly localized to the nuclei as the infection progressed. Finally, NF-κB completely overlapped with the cell nuclei at 24 h p.i. NF-κB molecules also increased in the nuclei of TC0668^mut^-infected cells, but some green fluorescence was still maintained outside the nuclei and it only partially overlapped with the cell nuclei at 24 h p.i.

### Inflammation- and Fibrosis-Related Proteins Differentially Expressed Between TC0668^wt^- and TC0668^mut^-Infected HeLa Cells

Tables 2, 3 show inflammation- and fibrosis-related proteins with differential expression (*P* < 0.05) between TC0668^wt^- and TC0668^mut^**-**infected cells at 18 h p.i. A total of 36 pro-inflammatory proteins and 27 fibrosis-related proteins presenting a clear fold-change (TC0668^mut^/TC0668^wt^) of ≥1.2 or ≤ 0.83 are shown with functional annotation, and they may play roles in chlamydial pathogenesis.

**Table 2:** Differentially expressed proteins related with inflammation in TC0668^mut^- vs. TC0668^wt^-infected HeLa cells at 18 h p.i.

**Accession no**.	**Protein name**	**Gene** **name**	**iTRAQ** **ratio**
IF16_HUMAN^a^	Gamma-interferon-inducible protein 16	IFI16	0.547155
A0A192GQE0_HUMAN	Caspase recruitment domain-containing protein 11	CARD11	1.47179
Q8IZA9_HUMAN	Cyclooxygenase 2b	N/A	0.595394
Q99557_HUMAN	NF-IL6	N/A	0.686745
M1XK38_HUMAN	MHC class I antigen	HLA-A	1.291266
S6AP35_HUMAN^a^	MHC class II antigen	HLA-DQB1	1.500968
F8W696_HUMAN	Apolipoprotein A-I	APOA1	1.319977
B3KRK2_HUMAN	DEAD (Asp-Glu-Ala-Asp) box polypeptide 41, isoform CRA a	DDX41	1.326954
HERC5_HUMAN	E3 ISG15–protein ligase HERC5	HERC5	0.708278
B3KNA9_HUMAN	cDNA FLJ14150 fis, clone MAMMA1003026, highly similar to Probable ubiquitin carboxyl-terminal hydrolase CYLD	N/A	1.340283
L8B4J3_HUMAN	Ubiquitin C	UbC	0.371092
A0A1D0C403_HUMAN	MHC class I antigen	HLA-C	1.288396
E7DRJ7_HUMAN	MHC class I antigen	HLA-B	1.297405
MAPK2_HUMAN^a^	MAP kinase-activated protein kinase 2	MAPKAPK2	0.44963
TSP1_HUMAN^a^	Thrombospondin-1	THBS1	1.275128
C9J102_HUMAN	Histone deacetylase 7	HDAC7	0.656532
E9PHH9_HUMAN	DNA-directed RNA polymerase III subunit RPC3	POLR3C	0.763347
MCP_HUMAN	Membrane cofactor protein	CD46	1.341189
A8K6K4_HUMAN	cDNA FLJ77565, highly similar to Homo sapiens interleukin 1 receptor accessory protein (IL1RAP), transcript variant 1, mRNA	N/A	1.312878
H0YAS8_HUMAN	Clusterin	CLU	0.70595
A0A087WYV6_HUMAN	Tetraspanin-6	TSPAN6	0.729155
SHIP1_HUMAN	Phosphatidylinositol 3,4,5-trisphosphate 5-phosphatase 1	INPP5D	1.422254
A0A0J9YY65_HUMAN	Alpha-2-antiplasmin	SERPINF2	1.290964
B7Z8S4_HUMAN	cDNA FLJ55316	N/A	1.321434
PSB4_HUMAN	Proteasome subunit beta type-4	PSMB4	0.765327
PEDF_HUMAN	Pigment epithelium-derived factor	SERPINF1	1.404227
MK07_HUMAN	Mitogen-activated protein kinase 7	MAPK7	0.757829
SHRPN_HUMAN	Sharpin	SHARPIN	0.71324
B4E2Y1_HUMAN	cDNA FLJ52879, highly similar to Peroxisome proliferator-activated receptordelta	N/A	1.404332
A2MG_HUMAN	Alpha-2-macroglobulin	A2M	1.39679
A0A087WSY5_HUMAN	Carboxypeptidase B2	CPB2	0.624017
A8K2T4_HUMAN	cDNA FLJ78207, highly similar to Human complement protein component C7 mRNA	N/A	1.307322
B7Z8Q7_HUMAN	cDNA FLJ53871, highly similar to Inter-alpha-trypsin inhibitor heavy chain H4	N/A	1.431388
SMAD1_HUMAN	Mothers against decapentaplegic homolog 1	SMAD1	0.622682
Q9HBQ7_HUMAN	Cathepsin L, isoform CRA_b	CTSL	0.737747
TRAD1_HUMAN^a^	TRAF-type zinc finger domain-containing protein 1	TRAFD1	0.484745

Differentially expressed proteins in HeLa cells infected with C. muridarum TC0668^wt^- or TC0668^mut^-strains at 18 h p.i. For all proteins, only fold-changes above 1.2-fold or below 0.8-fold and with a P-value of < 0.05 (using t-test) for changes in expression between TC0668^wt^- and TC0668^mut^-infected HeLa cells are shown.

a *qRT-PCR validation was performed with the protein*.

**Table 3:** Differentially expressed proteins related with fibrosis in TC0668^mut^- vs. TC0668^wt^-infected HeLa cells at 18 h p.i.

**Accession no**.	**Protein name**	**Gene** **name**	**iTRAQ** **ratio**
B4DHJ4_HUMAN	cDNA FLJ57931, highly similar to MORC family CW-type zinc finger 3	N/A	0.752665
TSP1_HUMAN^a^	Thrombospondin-1	THBS1	1.275128
A0A087WSY5_HUMAN	Carboxypeptidase B2	CPB2	0.624017
A0A0J9YY65_HUMAN	Alpha-2-antiplasmin	SERPINF2	1.290964
B7Z2X4_HUMAN	cDNA FLJ53327, highly similar to Gelsolin	N/A	1.306063
A6PVM9_HUMAN	Allograft inflammatory factor 1-like	AIF1L	0.75395
B3KT04_HUMAN	cDNA FLJ37385 fis, clone BRAMY2026405, highly similar to StAR-related lipid transfer protein 13	N/A	0.702884
A0A0A0MRF6_HUMAN	A-kinase anchor protein 9	AKAP9	1.284979
APC_HUMAN	Adenomatous polyposis coli protein	APC	0.742953
CTRO_HUMAN	Citron Rho-interacting kinase	CIT	0.718418
ACTBM_HUMAN	Putative beta-actin-like protein 3	POTEKP	0.428488
JMY_HUMAN	Junction-mediating and -regulatory protein	JMY	0.766594
B4DXH2_HUMAN	cDNA FLJ51138, highly similar to Arfaptin-2	N/A	1.552765
GMFB_HUMAN	Glia maturation factor beta	GMFB	1.352438
B2RA70_HUMAN	Tyrosine-protein kinase	N/A	1.308941
K22E_HUMAN	Keratin, type II cytoskeletal 2 epidermal	KRT2	2.019879
K1C14_HUMAN	Keratin, type I cytoskeletal 14	KRT14	2.181257
B4DJM5_HUMAN	cDNA FLJ61294, highly similar to Keratin, type I cytoskeletal 17	N/A	1.282977
F8W696_HUMAN	Apolipoprotein A-I	APOA1	1.319977
Q68E09_HUMAN	Uncharacterized protein DKFZp686N1969	DKFZp686N1969	0.750781
RPB1_HUMAN	DNA-directed RNA polymerase II subunit RPB1	POLR2A	1.287611
B4DKH9_HUMAN	cDNA FLJ51235, highly similar to Acidic fibroblast growth factorintracellular-binding protein	N/A	0.604194
L8B4J3_HUMAN	Ubiquitin C	UbC	0.371092
NR4A1_HUMAN	Nuclear receptor subfamily 4 group A member 1	NR4A1	0.526685
A2MG_HUMAN	Alpha-2-macroglobulin	A2M	1.39679
F2RM35_HUMAN	Serine protease	factor IX F9	1.282877
E9PIT3_HUMAN	Prothrombin	F2	1.330931

Differentially expressed proteins between C. muridarum TC0668^wt^- or TC0668^mut^-infected HeLa cells at 18 h p.i. have been analyzed for fibrosis. For all proteins, only fold-changes above 1.2-fold or below 0.8-fold and with P-value of < 0.05 (using t-test) for changes in expression between TC0668^wt^- and TC0668^mut^-infected HeLa cells are shown.

a *qRT-PCR validation was performed in this table for partial protein*.

As shown in Table 2, proteins that regulate type I interferon production (encoded by IFI16, DDX41, HERC5, UbC, HLA-A, HLA-DQB1, and POLR3C) and the CARD11 gene (encoding caspase recruitment domain-containing protein 11), which positively regulates NF-κB activity, were all more highly expressed in TC0668^wt^-infected cells. Nine proteins involved in interleukin (IL) production (encoded by IFI16, HLA-C, HLA-B, THBS1, MAPKAPK2, INPP5D, HDAC7, CD46, and APOA1) and four proteins that affect TNF production (encoded by IFI16, MAPKAPK2, CD46, and CLU) were also differentially expressed. Furthermore, multiple other differentially expressed proteins (encoded by TSPAN6, PSMB4, SERPINF1, MAPK7, SHARPIN, A2M, CPB2, SMAD1, CTSL, TRAFD1, and SERPINF2) were found to be related to inflammatory responses.

The differentially expressed fibrosis-related proteins in Table 3 include three proteins that are involved in the regulation of fibrinolysis (encoded by THBS1, CPB2, and SERPINF2), 11 proteins that participate in supramolecular fiber organization (encoded by AIF1L, AKAP9, APC, CIT, POTEKP, JMY, GMFB, KRT2, KRT14, APOA1, and DKFZp686N1969), three proteins that are associated with fibrin clot formation (A2M, factor IX F9, and F2), and three proteins that play important roles in the response to fibroblast growth factor (encoded by POLR2A, UbC, and NR4A1).

**Figure 8:**
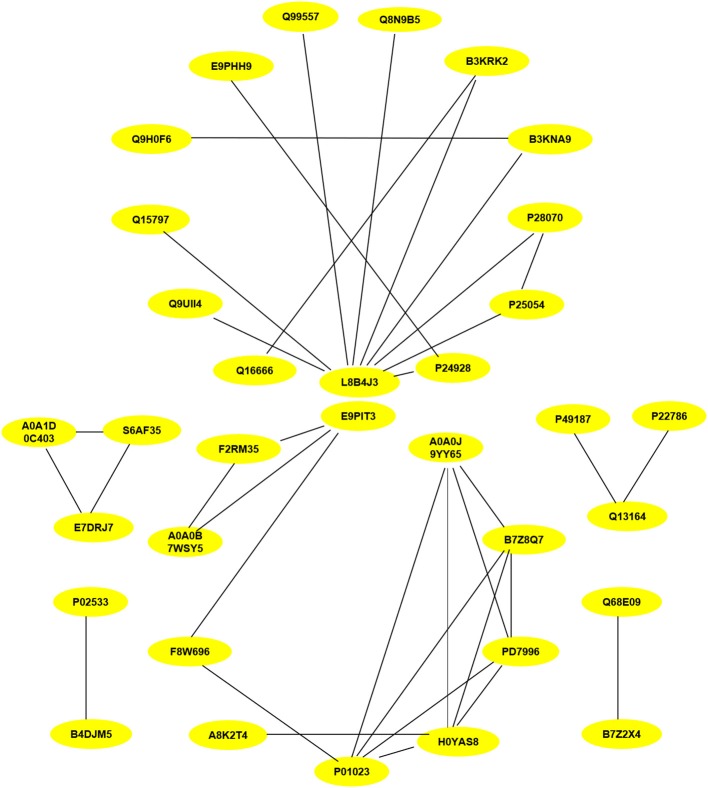
Protein-protein interaction network of statistically differentially expressed proteins (*P* < 0.05) associated with inflammation and fibrosis at 18 h p.i. The proteins interconnectivity of two categories (inflammation and fibrosis) are shown. The yellow circles marked with symbols combining numbers and letters, represent the protein ID of differentially expressed proteins screened by comparison of TC0668^mut^-infected and TC0668^wt^-infected HeLa cells. The corresponding information of proteins can be analyzed via UniProt (https://www.uniprot.org/), each protein ID corresponds to a protein. For example, the protein ID “P01023” corresponds to the protein “pha-2-macroglobulin”.

PPI assays were used to investigate the interaction between proteins, it will deepen the understanding of protein structure and function, especially that we are interested in. Therefore, the differentially expressed proteins (*P* < 0.05) related to inflammation and fibrosis, which might play important roles in *Chlamydia*-induced upper genital tract damage were employed in a PPIs network analysis using the STRING database. As shown in Figure 8, ubiquitin C (UbC) and alpha-2-macroglobulin (A2M), the most connected differentially expressed proteins, interacted directly with multiple proteins such as NF-IL6, cDNA FLJ14150, and cDNA FLJ53871. Proteins with high connectivity may be key points affecting the entire system of metabolism or signaling transduction pathways during chlamydial infection. Thus, a better understanding of the molecular activity of TC0668 can be gained by combining the results of PPI network analysis and GO annotations, which could facilitate further research on the molecular mechanisms underlying *C. muridarum* infections.

## Discussion

Studying protein function has become more relevant in the post-genomic era because the main embodiment of function in organisms is proteins. Additionally, the ability of naturally occurring plasmid-free chlamydial strains to establish infection drives the enthusiasm to investigate the role of chromosomal proteins in chlamydial pathogenesis (Kari et al., 2011; Sigar et al., 2014). Previous studies demonstrated that TC0668^mut^ strains are highly attenuated and cause less upper genital tract diseases in murine models compared to TC0668^wt^ strains (Chen et al., 2015; Conrad et al., 2016; Shao et al., 2017). The pathogenesis differences regarding *in vivo* infections between TC0668^mut^ and TC0668^wt^ strains suggest that TC0668 contributes to a robust infection and the induction of pathological inflammation (Chen et al., 2015; Conrad et al., 2016). However, the role of TC0668 in inducing inflammatory responses remains unclear. To better understand the role of TC0668 in the pathogenesis of *C. muridarum*, we investigated the biological functions of differentially expressed proteins associated with TC0668 expression using an iTRAQ-based quantitative proteomics analysis of HeLa cells infected with TC0668^wt^ or TC0668^mut^ strains.

HeLa cells have been widely used in chlamydial studies, which include mouse model genital pathogen *C. muridarum*, respiratory pathogen *Chlamydia psittaci* or *Chlamydia pneumonia* (Bulir et al., 2015; Koch-Edelmann et al., 2017; Gallegos et al., 2018). Most importantly, we have conducted some *in vitro* experiments both in the murine oviduct cell C57epi.1 and human cervical cell line HeLa, and found the results are consistent with each other (unpublished data). It's similar to previous study that both murine cells BM12.4 and human epithelial cells HeLa 229 infected with different chlamydial strains (*C. trachomatis* serovars A to H, L1 to L3, *C. muridarum, C. pneumonia*, and *C. caviae*) exhibited the same protein tyrosine phosphorylation patterns (Virok et al., 2005). It's practicable to apply human HeLa cells in our research.

Molecular databases have long been a powerful tool for the study of molecular evolution and the prediction of protein function. The iTRAQ results suggest that TC0668 influences many important proteins that are molecular switches involved in cell signal transduction and biological activity. Ten differentially expressed proteins (encoded by MAPKAPK2, TRAFD1, IFI16, SRPRB, JAK1, PMM1, HLA-DQB1, THBS1, ITPR1, and BCAP31) were selected for qRT-PCR analysis to validate the proteomic results using qRT-PCR, and the qRT-PCR results were consistent with the proteomics results. Hence, iTRAQ-based techniques can be key in proteomics analysis, potentially providing useful information to help to reveal regulatory mechanisms.

Protein function prediction and identification are useful to clarify mechanisms underlying changes due to specific physiological or pathological conditions. GO and KEGG analyses are often performed to investigate the potential biological roles of differentially expressed proteins (Zou et al., 2018; Cai et al., 2019). With regard to GO, the differentially expressed proteins were mainly involved in biological processes including cellular process, metabolic process, immune system process, and biological regulation, suggesting that the pathogenesis of TC0668^wt^ strains might be regulated by complex signaling pathways. KEGG analysis showed that these differentially expressed proteins are significantly enriched in disease-related signaling pathways such as the PI3K/Akt and NF-κB signaling pathways. PI3K is a lipid kinase that phosphorylates inositol phospholipids, thereby controlling membrane lipid composition and regulating a range of processes within the cell, including vesicle transport and signal transduction (Fruman et al., 2017). Western blotting involving key molecules of the PI3K/Akt pathway showed that PI3K and p-Akt expression was significantly higher in TC0668^wt^-infected cells than TC0668^mut^-infected cells at all time points tested. As one of the effector molecules of the PI3K signaling pathway, p53 can repress genes involved in cell growth stimulation and regulate normal cell activities by mediating signal transduction. In contrast to PI3K and p-Akt expression, p53 expression was significantly lower in the TC0668^wt^-infected cells compared to the TC0668^mut^-infected cells. Previous research has shown that *Chlamydia* infection of HeLa cells promotes p53 degradation (Bensaad et al., 2006), which is consistent with the results of our study. Additionally, stable p53 expression in HeLa cells interferes with the growth of *Chlamydia* by regulating the energy metabolism of the host cells, thereby inhibiting the infection (Siegl et al., 2014). We therefore hypothesized that the TC0668^wt^ strain may facilitate the degradation of p53 by activating the PI3K/Akt signaling pathway, thereby relieving the inhibition of chlamydial growth, promoting infection, and further enhancing chlamydial pathogenicity. Further studies involving the TC0668^mut^ and TC0668^wt^ strains are needed to address the effects of the PI3K/Akt-p53 axis on chlamydial growth and metabolism.

The NF-κB pathway is a ubiquitous and classical pathway responsible for mediating DNA transcription, innate and adaptive immunity, inflammation, and other cellular activities (Bakkar and Guttridge, 2010). In general, it exists freely in the cytoplasm, inhibited by IκB proteins. With a variety of stimuli, such as cellular stress or bacterial infection, activated NF-κB translocates to the nucleus and induces target gene expression, exerting transcriptional regulation. In our western blotting analysis of the NF-κB pathway, NF-κB (p65) expression increased over time as the infection progressed in both TC0668^wt^- and TC0668^mut^-infected cells, but NF-κB (p65) levels in TC0668^wt^-infected cells were significantly higher than those in TC0668^mut^-infected cells at all time points (*P* < 0.05). IFAs were used to further detect the translocated NF-κB in nuclei at different time points in the two groups of cells. It was found that, for the TC0668^wt^ strain at 24 h, NF-κB fluorescence signals completely overlapped with the cell nuclei, whereas some NF-κB fluorescence signals were still located outside the nuclei at 24 h for the TC0668^mut^ strain. Therefore, we hypothesized that TC0668 is involved in regulating the activation of the NF-κB transcription factor, thereby mediating the inflammatory response to *C. muridarum*.

Inflammation is considered one of the most common consequence of persistent chlamydial infections (Cheong et al., 2018; Hou et al., 2018; Jia et al., 2019). To investigate the potential roles of TC0668 in inducing inflammatory- and fibrosis-related pathogenesis, a PPI network analysis of differentially expressed proteins at 18 h p.i was performed. It is worth noting that the expression levels of various proteins related to the activities of chemokine, IL (such as IL-6 and IL-1), tumor necrosis factor and associated cytokines, and type-1 interferon were significantly different between TC0668^wt^- and TC0668^mut^-infected cells. Ubiquitin C (UbC), a highly connected protein, has been shown to be involved in several diseases and to mediate biological processes such as inflammation, programmed cell death, and proliferation (Chen et al., 2008). In addition, alpha-2-macroglobulin (A2M), another highly connected protein, plays a pivotal role in eukaryote innate immune responses by binding to and modulating biological molecules (James, 1980). Thus, these two highly connected proteiin may play key roles in triggering, regulating, and modifying cell signaling transduction related to TC0668-mediated biological processes during chlamydial infection.

At present, the prediction of protein function is a challenging problem in the study of PPI networks. However, our findings suggest that TC0668 induces proteins to trigger, modify, and regulate signaling pathways that lead to pathogenesis, and the findings therefore provide new insights into the pathogenicity and potential molecular signaling pathways of *C. muridarum*. The differential effects on PI3K and NF-κB signaling pathways may underlie the attenuated virulence exhibited by the TC0668^mut^ strain compared to the TC0668^wt^ strain to induce hydrosalpinx.

## Conclusions

As a chlamydial protein, TC0668 participates in the induction of inflammation, fibrosis, metabolic processes, and other cellular activities by regulating various molecular responses and signaling pathways, playing important roles in the pathogenesis of *C. muridarum*. This study provides useful information on the role of TC0668 in *C. muridarum* pathogenicity.

## Data Availability Statement

The raw data supporting the conclusions of this manuscript will be made available by the authors, without undue reservation, to any qualified researcher.

## Author Contributions

YW: data preparation and interpretation, and writing the manuscript. EA: modifying the manuscript. NL: cell experimentation. XL: western blotting experiments. WX: extracting the proteins. AM: participation in language editing. ZL: intellectual contribution throughout the study. ZZ: intellectual contribution throughout the study and interpretation of data. All authors reviewed, read, and approved the final manuscript.

### Conflict of Interest

The authors declare that the research was conducted in the absence of any commercial or financial relationships that could be construed as a potential conflict of interest.

## Supplementary Material

Supplementary Table 1 The differentially expressed proteins between TC0668^wt^- and TC0668^mut^-infected cells in Venn diagram.
